# Effect of Finishing Protocol on Obtaining and Maintaining Gloss in Four Nanohybrid Resin Composites

**DOI:** 10.7759/cureus.69073

**Published:** 2024-09-10

**Authors:** Julissa Marín-Velasquez, Laura Osorio-Vélez, Lippo Lassila, Carlos M Ardila

**Affiliations:** 1 Prosthodontics Postgraduate Program, University of Antioquia, Medellín, COL; 2 Biomaterials Science, Turku Clinical Biomaterials Centre, University of Turku, Turku, FIN; 3 Basic Sciences Department, Faculty of Dentistry, University of Antioquia, Medellín, COL

**Keywords:** aging, dental finishing, dental polishing, gloss, nanohybrid resin composites

## Abstract

Objective: Within the clinical objectives of performing direct restorations with resin composites is the achievement of an optimal surface in mechanical terms, while also resembling the natural optical characteristics of dental enamel. The aim of this study is to compare the gloss levels achieved in four nanohybrid resins using four different finishing/polishing (F/P) protocols and assess the retention of gloss after an aging process.

Methods: Forty-eight A2-colored samples from four commercial brands (Filtek Z250 XT, Tetric N-Ceram, Zafira, and Spectra Smart) were prepared, with 12 specimens per resin. The samples were divided into four subgroups based on the F/P treatment: T1 (clinical polishing sequence with ShapeGuard system), T2 (metallographic polishing sequence), T3 (clinical polishing sequence with PermaSeal), and T4 (final polymerization through glycerin and clinical polishing sequence). Gloss measurements were taken before and after aging. ANOVA one-way was done.

Results: Before aging, Z250 showed the highest average gloss in T1 (77.2 gloss units - GU), while Zafira (43.8 GU), Tetric (39.5 GU), and Spectra (33.5 GU) had lower values. In T2, Zafira (92.7 GU) and Spectra (87.4 GU) had the highest average gloss, and Tetric (75.7 GU) and Z250 (78.3 GU) had the lowest. No significant differences were found in T3, and in T4, Z250 had the highest average gloss (78.9 GU), followed by Tetric (74.7 GU) and Zafira (52.5 GU), with Spectra having the lowest (42.7 GU). Control group gloss levels ranged from 61.8 to 71.8 GU and remained consistent after aging, except for Tetric in T1 (p=0.045).

Conclusions: The impact of F/P strategies on gloss retention varies with the material, even within the same resin composite classification. The initial gloss level appears to be a more critical factor for long-term gloss retention than the specific F/P protocol used.

## Introduction

Achieving optimal mechanical properties and natural esthetics are key objectives in direct restorations with resin composites. Nanohybrid resins, known for their strong chemical integration of nanoparticles and easy polishability due to reduced filler particle size, are widely chosen for anterior restorations due to their balanced mechanical and esthetic properties [1,2].

The esthetic quality of resin composites is primarily assessed based on texture, color, and gloss, as these dimensions significantly impact clinical outcomes [2]. A rough surface not only reduces gloss by causing random light dispersion but also favors bacterial growth, highlighting the importance of effective finishing and polishing for the longevity and appearance of restorations [1,2].

Finishing involves adjusting the contour to achieve proper anatomy, while polishing removes roughness and scratches using abrasive tools [3]. Metallographic polishing, employing silicon carbide papers and alumina paste, is effective in enhancing gloss [4]. Surface sealants, designed to penetrate microdefects, are another method used to optimize final finishing [5].

During resin polymerization, an oxygen-inhibited layer forms on the surface, leading to a polymer chain prone to staining and wear [6]. Methods like covering the resin with glycerin during light activation have been proposed to inhibit this layer, enhancing the resin’s mechanical and esthetic properties [2,7].

Specular gloss, a parameter influenced by material surface properties and measured at angles of 20º, 60º, and 85º, is an important indicator of surface quality [8]. A 60º angle is standard for moderate gloss values, aligning closely with the average observer’s perspective [9,10]. Gloss is quantified by the ratio of the detector signal to that of a reference material, typically polished black glass with a refractive index of n=1.567 [11].

This study aims to evaluate the influence of different finishing and polishing protocols on the initial gloss attainment and long-term gloss retention of four nanohybrid resins. The first hypothesis posits that there are no statistically significant differences in the initial gloss attainment among the four nanohybrid resins when subjected to different finishing and polishing protocols. The second hypothesis asserts that there are no statistically significant differences in gloss retention after aging among the resins, regardless of the finishing and polishing treatments applied.

## Materials and methods

Based on a previous study that found statistically significant differences in gloss among four types of resins [2] and considering an alpha of 0.05 and a power of 80%, a sample size of at least 12 specimens per group was established. Thus, 48 samples were prepared using nanohybrid resins from four commercial brands in the A2 shade: Filtek Z250 XT (3M ESPE, Minnesota, US), Tetric N-Ceram (Ivoclar Vivadent AG; Schaan, Liechtenstein), Zafira (New Stetic, Antioquia-Colombia), and Spectra Smart (Dentsply Sirona Inc, North Carolina, US). The sample size and power were defined considering the proposed objective. For each material type, 12 samples were made using metallic molds with a thickness of 2 mm and a diameter of 8 mm. These molds were placed on a Mylar strip and a glass slide. Then, the sample was placed on a support designed to standardize the LED lamp position in the three spatial axes, at a perpendicular distance of 1 mm. The photopolymerization was performed by using a Bluephase N LED curing light (Ivoclar Vivadent AG, Schaan, Liechtenstein) with an irradiance of 1,200 mW/cm^2^ for 30 seconds on each sample. All samples were immersed in distilled water and stored for 24 hours at a temperature of 37°C in an incubator (model BPN-80CH, CMLab). The specimens were categorized into two sides: Side A: the surface where photocuring, finishing, and polishing were performed, and Side B, which is the intragroup control surface. Gloss measurements were taken before and after aging, differentiating control and treated surfaces with labels placed on the specimens. Subsequently, the samples were distributed into four subgroups based on the finishing and polishing (F/P) treatment using simple random sampling. This process was carried out by the same blinded operator. Materials are detailed in Table 1.

**Table 1:** Composition of materials Bis-GMA: bisphenol A glycidyl methacrylate; Bis-EMA: ethoxylated bisphenol A dimethacrylate; PENTA: dipentaerythritol penta-acrylate monophosphate; TEGDMA: triethylene glycol dimethacrylate; UDMA: urethane dimethacrylate; DMAEMA: 2-dimethylaminoethyl methacrylate; PEDMA: poly (ethylene glycol) dimethacrylate; BAGB: barium aluminum borosilicate; BAFG: barium aluminum fluoroborosilicate; Ɣ-MPS: Gamma-Methacryloxypropyltrimethoxysilane; NR: not reported; NA: not applicable.

Material	Manufacturer	Organic Matrix	Filler Type	Average Particle Size	Particle Shape	Filler Weight %	Filler Volume%
Z250 XT	3M ESPE	Bis-GMA, UDMA, BIS-EMA, PEGDMA and TEGDMA	Zirconia/silica	20 nm, < 3µm	Spherical, nanoclusters	82	68
Tetric N-Ceram	Ivoclar Vivadent AG	Bis-GMA, UDMA, TEGDMA	Silica, barium glass, ytterbium trifluoride, silicon dioxide, and mixed oxide	40 nm - 7 µm	NR	82	65
Zafira	New Stetic	Bis-GMA, TEGDMA, UDMA, BIS-EMA	Barium glass and silicon dioxide, Ɣ-MPS, BAFG, BABG	40 nm - 2 µm	Spherical	75 - 78	55 - 60
Spectra Smart	Dentsply Sirona	Bis-GMA modified with urethane, TEGDMA	BABG, BAFG, silica nanoparticles	10 - 20 nm, 0.7 - 0.9 µm	NR	75 - 77	58
PermaSeal	Ultradent Products, Inc.	Bis-GMA, TEGDMA, DMAEMA		NA	NA	NA	NA
Liquid Strip	Ivoclar Vivadent AG	Glycerin, water, high-dispersion silica, and high-dispersion aluminum oxide		NA	NA	NA	NA

Treatment 1 (T1): Clinical polishing sequence

Manual wet polishing was performed for 20 seconds using Abracol silicon carbide paper (FEPA) with grit sizes of 280, 600, and 1500, simulating the clinically used finishing burs [12]. For polishing and getting the final gloss, a W&H electric motor (Bürmoos, Austria) was used with a horizontal, unidirectional motion using ShapeGuard polishers (Diatech-Coltene Altstätten, Switzerland.) under microscopy Omni Pico Zeiss (0.6× magnification) (Jena, Germany). The first step was made with a medium-grit polisher equivalent to 32-69 µm (FEPA 360-220) for 20 seconds, the second one, by a superfine-grit polisher equivalent to 4-8 µm (FEPA 1000) for 20 seconds at 10,000 rpm (according to manufacturer instructions) [4].

Treatment 2 (T2): Metallographic polishing sequence and aluminum oxide

Manual wet polishing was performed using Abracol silicon carbide paper (FEPA) with grit sizes of 280, 600, 1500, and 2000 for 20 seconds. Subsequently, a cloth and 0.3 µm aluminum oxide were used at 500 rpm with gentle pressure for 20 seconds [4].

Treatment 3 (T3): Clinical polishing sequence and PermaSeal

The same protocol as in T1 for clinical polishing was followed, and then the surface sealant PermaSeal (Ultradent Products Inc., Utah, US) was applied following the manufacturer’s instructions. Specifically, all composite surfaces to be sealed were etched for 5 seconds with 35% phosphoric acid, then rinsed and dried thoroughly. A thin layer of PermaSeal sealer was then applied to the surface of the composite, gently air-thinned, and cured for 10 seconds.

Treatment 4 (T4): Glycerin polymerization and clinical polishing sequence

Before polymerizing the final increment of resin composite, the surface was coated with a droplet of Liquid Strip (Ivoclar Vivadent AG, Shaan, Liechtenstein) following manufacturer instructions, and light activation occurred through this layer. Subsequently, the excess material was removed using pressurized water. The same sequence as T1 was then repeated.

**Figure 1:**
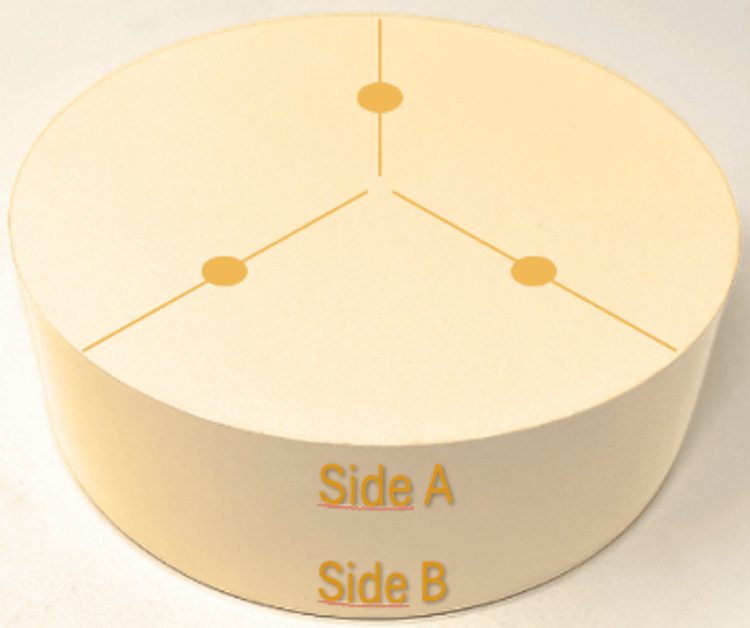
Diagram illustrating the measurement points for gloss values on the sample. The three measurement points are located at the center of each sample, on both sides, as identified by lateral marks. These points were used to establish a baseline for gloss values, ensuring consistent measurement across all samples.

To determine the gloss value, a glossmeter was used, expressing the results in gloss units (GU) [13]. A baseline for gloss values was initially established, employing three measurement points at the center of each sample on both sample sides (identified by lateral marks) (Figure 1).

This was achieved using a calibrated Zehntner-Glossmeter infrared device (GmbH Testing Instruments, Darmstadt, Germany) with a 60° angle of incidence.

The aging process was carried out in distilled water at a temperature of 55°C for four days (samples weighed before and after) and three gloss measurements were taken at the center of each sample on both sides again. Glossmeter characterization was performed in the biomaterials laboratory, ensuring impartiality and controlling observer bias.

Statistical analysis

An IBM® SPSS program (version 27) was utilized for data analysis. A univariate descriptive analysis including summary measures such as the mean and standard deviation accompanied by confidence intervals was conducted to evaluate the quantitative variable, i.e., gloss measured in GU. Subsequently, a bivariate analysis using one-way ANOVA was performed.

When the ANOVA test indicated statistically significant differences, multiple range analyses (post hoc) were applied. Depending on the equality or difference of variances between groups, Bonferroni or Games-Howell methods were employed, respectively. A comparison of gloss before and after the aging process was conducted using the student’s t-test for repeated samples. The results with a p-value < 0.05 were considered as significant.

## Results

The statistical results for the average gloss, along with their standard deviation and 95% confidence intervals, are presented in Table 2.

**Table 2:** Summary measures by resin composite and polishing system before aging X±SD=mean±standard deviation; a p-value < 0.05 indicates a statistically significant difference, based on a one-way ANOVA test; 95% CI: 95% confidence interval; GU: gloss units. Identical uppercase letters indicate no significant difference among treatments within the same resin, while different uppercase letters signify a significant difference among treatments within the same resin. Identical lowercase letters indicate no significant difference in the same treatment among the four resins, while different lowercase letters signify a significant difference in the same treatment.

		Resins				
Treatments		Spectra (GU)	Tetric (GU)	Z250 XT (GU)	Zafira (GU)	p-value
Control	X±SD	71.8 ± 15.5 ^a^	70.0 ± 8.4 ^a^	61.8 ± 10.3 ^a^	66.6 ± 11.0 ^a^	0.174
	95% CI	62.0 – 81.7	64.6 – 75.3	55.2 – 68.3	59.5 – 73.6	
Clinical polishing	X±SD	33.5 ± 5.7 ^Aa^	39.5 ± 5.0 ^Aa^	77.2 ± 3.4 ^Ab^	43.8 ± 12.3 ^Aa^	0.0001
	95 % CI	19.4 – 47.6	27.1 – 51.8	68.8 – 85.7	13.2 – 74.5	
Metallographic polishing	X±SD	87.4 ± 1.7 ^Ba^	75.7 ± 11.1 ^Bab^	78.3 ± 1.6 ^Ab^	92.7 ± 3.8 ^Ba^	0.028
	95 % CI	83.2 – 96.6	48.0 – 103.3	74.3 – 82.4	83.2 – 102.1	
Clinical polishing+ PermaSeal	X±SD	45.3 ± 11.9 ^Aa^	43.9 ± 9.3 ^Aa^	53.6 ± 14.0 ^Aa^	55.5 ± 7.1 ^Aa^	0.500
	95 % CI	15.8 – 74.8	20.9 – 67.0	18.9 – 88.3	37.9 – 73.1	
Glycerin + clinical polishing	X±SD	42.7 ± 10.0 ^Aa^	74.7 ± 9.3 ^Bb^	78.9 ± 20.4 ^Aab^	52.5 ± 14.2 ^Aab^	0.039
	95 % CI	17.9 – 67.5	51.5 – 97.9	28.3 – 129.6	17.1 – 87.9	
p-value		0.0001	0.001	0.101	0.002	
F value one-way ANOVA test		14.13	12.35	2.35	6.43	

Before aging (By F/P protocol)

In T1, it was observed that the Z250 XT resin composite exhibited the highest average gloss (X̄ 77.2 GU), along with the lowest standard deviation. Conversely, the Spectra Smart resin composite obtained the lowest gloss value (X̄ 33.5 GU). However, no significant differences were found between the Spectra Smart, Tetric N-Ceram, and Zafira composite disks.

In T2, the Zafira resin composite displayed the highest average gloss (X̄ 92.7 GU). In this treatment, all values exceeded 75 GU, with the lowest average value recorded in Tetric N-Ceram (X̄ 75.7 GU).

In the group with PermaSeal (T3), no statistically significant differences were found in gloss among the four evaluated resins. However, significant differences were observed with other treatments and the control group for all resins. Additionally, this group exhibited the lowest gloss values in the study.

In the glycerin-based finishing treatment (T4), a higher average gloss was obtained for the Z250 XT resin composite (78.9 GU). However, this resin composite had also the highest standard deviation (20.4), and the Spectra resin composite recorded the lowest average gloss level (42.7 GU).

Before aging (By resin)

Upon analyzing differences for each resin composite group concerning the polishing system used, it was found that the only resin composite without statistically significant differences (p = 0.101) regarding the polishing treatment was Z250 XT. This resin composite exhibited the highest gloss values in all four evaluated treatments, with no statistically significant differences among them.

In the case of the Spectra Smart resin composite, it showed the lowest gloss values in all treatments (p > 0.05), except in the metallographic polishing protocol (87.4 GU).

It is essential to highlight that the Tetric N-Ceram resin composite did not show significant differences in gloss values, both in the clinical polishing treatment and the use of the surface sealant.

For Zafira resin, gloss values obtained through clinical polishing, the use of surface sealant, and the application of glycerin did not show statistically significant differences among them. However, metallographic polishing resulted in a significantly higher gloss value in the study (92.7 GU).

After aging

Table 3 depicts the summary measures by resin composite and polishing system, after the aging process.

**Table 3:** Comparison of gloss by resin composite and polishing system. Before and after the aging process X±SD: mean±standard deviation; a p-value < 0.05 indicates statistically significant differences, based on the paired t-Student test; GU: gloss units; BA: before aging; AA: after aging.

Resin Treatment		Spectra (GU)	Tetric (GU)	Z250 XT (GU)	Zafira (GU)
		X±SD	X±SD	X±SD	X±SD
Control	BA	71.8 ± 15.5	70.0 ± 8.4	61.8 ± 10.3	66.6 ± 11.0
	AA	75.4 ± 9.7	67.0 ± 6.3	66.7 ± 13.4	70.4 ± 13.2
	p-value	0.192	0.057	0.076	0.043
Clinical polishing	BA	33.5 ± 5.7	39.5 ± 5.0	77.2 ± 3.4	43.8 ± 12.3
	AA	25.6 ± 6.5	49.0 ± 8.5	76.2 ± 9.4	34.8 ± 9.5
	p-value	0.247	0.045	0.893	0.093
Metallographic polishing	BA	87,4 ± 1,7	75.7 ± 11,1	78.3 ± 1.6	92.7 ± 3.8
	AA	84.3 ± 2.2	76.1 ± 8.8	79.0 ± 2.3	90.7 ± 4.3
	p-value	0.068	0.849	0.218	0.139
Clinical polishing + PermaSeal	BA	45.3 ± 11.9	43.9 ± 9.3	53.6 ± 14.0	55.5 ± 7.1
	AA	42.9 ± 10.2	47.9 ± 9.8	53.5 ± 3.8	57.2 ± 18.4
	p-value	0.786	0.316	0.986	0.887
Glycerin + clinical polishing	BA	42.7 ± 10.0	74.7 ± 9.3	78.9 ± 20.4	52.5 ± 14.2
	AA	38.5 ± 3.9	56.6 ± 12.2	78.6 ± 11.4	54.8 ± 14.3
	p-value	0.652	0.071	0.953	0.760

In Table 3, comparisons between results before and after the aging process are presented. It can be observed that there is no significant difference in the gloss level in the treatments performed for each type of resin composite before and after aging, apart from Tetric N-Ceram in T1. In this case, there was a significant increase from 39.5 GU before aging to 49.0 GU after aging. In the control group (Mylar strip), gloss measures range from 66.7-75.4 before aging and 61.8 GU-71.8 GU after aging without significant difference.

## Discussion

We investigated the influence of four F/P protocols on the achievement and preservation of gloss in four distinct types of nanohybrid resins. To get the best esthetic result for dental composites, finishing and polishing are crucial for getting a clinically acceptable gloss, which contributes to the longevity of these types of restorations.

Upon scrutinizing the experimental findings, we observed statistically significant differences in gloss among the four resins subjected to the various F/P treatments, thereby rejecting the first hypothesis. Regarding the gloss permanence, no significant differences supporting one F/P treatment over another could be demonstrated for the Zafira, Z250XT, and Spectra Smart resins. Thus, the hypothesis was accepted. The significant variation in the Tetric N-Ceram + clinical polishing group may be paradoxical.

In restorative dentistry, with regard to polishing protocols, the goal is to achieve a level of gloss comparable to the enamel of natural teeth. The American Dental Association (ADA) has established as an ideal surface gloss that ranges between 40 and 60 GU [14]. Similarly, as it is the case for methacrylate composites, an acceptable gloss for dental ceramics has been stated in the range of 50-57 GU [15]. In our study, T1 was the only finishing system where results below 40 GU were obtained for the Spectra Smart resin composite (33.5 GU) and Tetric N-Ceram resin composite (39.5 GU). Meanwhile, T2 was the only F/P protocol that achieved a gloss level above 60 GU, with values as follows: Tetric N-Ceram 75.7 GU, Z250XT 78.3 GU, Spectra Smart 87.4 GU, and Zafira 92.7 GU. The control side exhibited a range of 61.8-71.8 GU, consistent with results reported by Soliman et al. (51.7 - 76.5) [16] and Lassila et al. (77-94 GU) [17]. While this gloss value surpassed that of T1, T3, and T4, it does not necessarily represent an optimal outcome due to impracticality on certain clinical surfaces (e.g., occlusal surfaces) and the risk of exceeding the natural gloss of enamel, potentially resulting in an artificial appearance of the restoration.

The Z250 XT and Tetric N-Ceram resins demonstrate a higher filler content compared to the other four evaluated resins. Z250 XT exhibits significantly higher brightness values, which can be attributed to the size of its filler particles ranging from 20 nm to 3 µm. Conversely, Tetric N-Ceram has a particle size range of 40 nm to 7 µm. It is essential to consider the Ytterbium (Tetric N-Ceram-Ivoclar) and Zirconia (Z250XT-3M) contents for gloss, given their high refractive indices (2.1750 and 1.5067, respectively). The elevated gloss observed for Z250 XT may also be attributed to a more uniform particle distribution and reduced separation between inorganic nanoclusters because the presence of small filler particles throughout the material protects the resinous matrix, which is softer than the fillers. This matrix is more susceptible to deterioration [15-17], which due to surface alterations can result in a lower reading in the glossmeter because of a lower light reflection.

Additionally, Zafira achieves gloss levels resembling dental enamel (43.8 GU), as per ADA-defined gloss parameters [14], while Spectra Smart falls outside the suggested range (33.5 GU). This discrepancy may be due to Spectra Smart’s particle size and lower filler content, leading to increased matrix disruption during polishing and diminished light reflection. Jassé’s study [18] supports the positive correlation between filler percentage and resin composite brightness, indicating that higher filler loads are associated with increased gloss both before and after aging. The particle shape is also considered as one of the most important aspects influencing material behavior [19]. Resin composites with spherical fillers, such as Z250 XT and Zafira, tend to show a higher degree of gloss and smoothness compared to irregular fillers [3].

On the other hand, the abrasive particles in a polishing system must be harder than the filler materials in resin composites. During finishing and polishing, the resin composite matrix is initially removed, leaving the harder filler particles in contact with the surface, enhancing the resin’s resistance to degradation. Polishing systems with aluminum oxide particles, like the ShapeGuard system (Diatech-Coltene), create smooth surfaces due to their superior hardness compared to resin composite fillers. Amaya-Pajares et al. [3] suggest that employing a polishing system and resin composite from the same manufacturer raises questions regarding whether similar and predictable polishing outcomes can be attained with polishers containing diamond or aluminum oxide particles, irrespective of the brand.

Regarding gloss in different types of resin composites, the results of Kaizer et al. [20] disagree with the classical conception of an inverse relationship between larger filler particle size and lower brightness. However, in the in vitro study by Zhang et al. [21], it was found that microhybrid resins were the only ones that failed to reach the clinical standard of 53 GU inspired by natural dental gloss [15]. On the other hand, de Melo et al. [22] suggest that the important benchmark to follow is >55 GU, which corresponds to an average roughness of 0.2 which is the critical point for bacterial retention based on a linear mixed-effects prediction mode.

Clinical polishing protocols, either alone or combined with glycerin and PS, involved using color-coded abrasive papers to simulate the order of burs in the finishing and polishing stages. The highest brightness in these samples correlates with the degree of abrasion, meeting ISO 6360 [23-26] standards for an ultrafine grain (>F1200). In contrast, ShapeGuard system polishers with lilac 523 (medium grain, 100-120 µm, between F360 and F220) and blue 503 (extra-fine grain, 30 µm, F1000) grades align with findings from Lassila et al.’s study [17]. Notably, samples subjected to clinical polishing alone (T1) did not meet the minimum required gloss (40 GU), possibly because of the assumption that polishing spirals continue the fine and ultrafine grain burs and it didn’t. With ShapeGuard, going backward in granularity compared to these burs may hinder the goal of eliminating roughness and scratches.

The treatment group with glycerin before polymerization may have been adversely affected by this subsequent clinical polishing, thus potentially experiencing the loss of the resin composite coverage that had polymerized better, except for Tetric N-Ceram, where the brightness value was significantly higher. The results, then, do not support a clear and direct relationship between the prior protection of polymerization and gloss of the restorations [6].

Similarly, in the study conducted by Mörmann et al. [15] is reported that the highest brightness level achieved on dental enamel through mechanical polishing was 53 GU. Additionally, in the study by Rocha et al. [8] was established that there were no differences in gloss perception from 50 GU onward. These results align with the study carried out by da Costa et al. [4], where gloss values between 0 and 40 were considered unacceptable.

It is noteworthy that, despite the reported benefits of utilizing surface sealants, as indicated by previous studies [5-7], our findings reveal that gloss values obtained through this approach were the lowest. These results may imply that surface sealants might not be conducive to achieving a glossy restoration. The outcomes of our study stand in contrast to those reported by Anagnostou et al. [24].

The degradation of resin composites, characterized by “detrimental changes in the chemical structure, physical properties, or appearance, resulting from the chemical cleavage of the macromolecules comprising them”[25], holds significant relevance in the field of dental biomaterials. Blumer et al.’s study has substantiated the influence of water immersion on polymers, thereby validating our decision to immerse the samples for four days at 55°C as a reliable aging method [26]. Due to their chemical structure, dimethacrylate resin composites are prone to water absorption which acts as a plasticizer, altering the interface between silane and filler particles and resulting in dimensional changes in the matrix, microcracks, roughness, and pigmentation [27].

In this study, the evaluated resins did not show statistically significant differences between F/P treatments in preserving gloss after undergoing an aging process, and differences between resins and treatments observed at baseline were maintained. This could be related to the small particle size and high filler content (>37%) combined with adequate silanization in all evaluated resins [25-27]. More evident effects on gloss are documented by brushing [23-25]. The paradoxical result of the Tetric N-Ceram group (T1), in which a significant increase in brightness was found after aging, may be related to the abrasiveness of the first step of the ShapeGuard polishers, which could leave the material more exposed to water absorption and filler protrusion to the surface, combined with the properties of Ytterbium.

Finally, it is relevant to emphasize that we have evaluated and compared four F/P protocols based on various strategies to achieve and maintain brightness. This differs from previous research where polishing instruments with alumina or diamond was used as comparative variable and roughness as response variable and without considering aging characterization [22-25]. The gloss parameter has been poorly studied but our work contributes to knowing how to get better esthetic and long-term performance in resin composites.

According to our results, the best protocol of F/P for every evaluated dental composite material would be: Z250XT (3M): acceptable gloss levels with any protocol of F/P tested here; Tetric Ceram (Ivoclar): Highest and consistent gloss level with metallographic F/P protocol, which indicates that is better a multistep polishers system with a progressively descendent grain size; Spectra Smart: Highest and consistent gloss level with metallographic F/P protocol, which indicates that is better a multistep polishers system with a progressively descendent grain size; Zafira (New Stetic): Highest and consistent gloss level with metallographic F/P protocol, which indicates that is better a multistep polishers system with a progressively descendent grain size; some variations in the protocols like longer polishment or combination with pastes could be advantageous for some of these materials and must be studied in other works.

A2 shade was chosen for all resin materials, as this is the standardized color parameter according to the Vita guide. Consequently, no further color standardization tests were conducted. While minor variations between brands may occur, no evidence suggests that this impacts gloss achievement [2,4].

The selection of a 60º angle for measuring specular gloss is supported by several standards, including ISO 2813, ASTMD 523, and DIN 67530, which identify this angle as a general standard for materials with moderate gloss values [9]. The rationale behind this choice is that it closely approximates the angle at which an average person perceives a surface’s gloss, making it a relevant metric for assessing clinical esthetics [10]. Additionally, the reflections observed at this angle are influenced by the material’s refractive index, as explained by Fresnel’s law of reflection [1,2]. This relationship between refractive index and gloss measurement is critical, as it directly impacts the accuracy and reliability of gloss indices obtained using standardized methods. The use of polished black glass with a refractive index of n=1.567 as a reference material ensures consistency in gloss measurements, facilitating comparisons across different studies and materials [11]. These aspects in the context of this study underscore the importance of selecting appropriate measurement angles and reference materials to accurately evaluate and compare the gloss properties of different resin composites.

Limitations

A limitation of this study is that surface roughness was not investigated to evaluate surface texture after finishing/polishing the resin composites. However, surface roughness can only be conducted in vitro, and this study aimed to replicate the polishing protocols that are conducted clinically. Therefore, surface gloss evaluation to assess whether restorations are properly polished is key.

Clinical studies that evaluate surface gloss and their perception on non-flat surfaces should be conducted. For surface roughness measurements, most studies involve the use of a profilometer. However, the inclusion of atomic force microscopy (AFM) measurements would be beneficial. The reason, the microroughness values obtained from AFM might be correlated with gloss values.

We strongly advocate for each resin composite manufacturing company to assess and disclose the optimal finishing and polishing (F/P) protocol aimed at consistently achieving a gloss within the range of 50-60 GU for each resin composite they produce. Such recommendations, encompassing details such as polisher composition, reduction in abrasive particle size, revolutions per minute, and duration, would contribute to the sustained retention of gloss, aligning with the findings of our study. Furthermore, future research could explore additional aspects, such as evaluating color changes before and after polishing, to provide a more comprehensive understanding of how F/P protocols influence not only gloss but also other esthetic properties of resin composites.

## Conclusions

Within the study’s limitations, we conclude that the finishing and polishing protocol significantly influences the gloss levels of resin composites. However, initial gloss does not necessarily predict long-term gloss retention after aging. Metallographic polishing, while effective in achieving higher gloss, has limited clinical applicability. Conversely, the simplified clinical sequence, although easier to implement, may compromise gloss retention over time. These findings highlight the need for clinicians to critically review and possibly adapt their polishing protocols to balance ease of use with long-term esthetic outcomes, emphasizing the importance of tailored approaches in achieving optimal and lasting results in direct restorations.
